# Diagnostic value and challenges in the immune regulation of pentraxin 3 in infectious diseases

**DOI:** 10.3389/fmicb.2026.1777246

**Published:** 2026-02-20

**Authors:** Teng-Hao Shao, Tie-Min Li, Jin-Wen Zhang, Xiao-Wei Lv, Xin-Tong Li, Na Cui, Ping Sheng

**Affiliations:** 1 Department of Intensive Care Unit, Affiliated Hospital of Hebei University, Baoding, China; 2 Department of Laboratory Medicine, Affiliated Hospital of Hebei University, Baoding, China; 3 Health Science Center, Hebei University, Baoding, Hebei, China

**Keywords:** biomarker, immune regulation, infectious diseases, inflammatory response, PTX3

## Abstract

Infectious diseases pose a severe threat to human health, and their early and precise diagnosis and intervention remain a major challenge in clinical practice. This review systematically examines the diagnostic value and immunomodulatory role of PTX3 across a spectrum of infectious diseases, including those affecting the respiratory, cardiovascular, digestive, urinary, and nervous systems, as well as orthopedic and skin infections. The aim is to provide novel perspectives for overcoming the bottleneck in precise diagnosis and treatment of infectious diseases. Currently, diagnosis and assessment primarily rely on clinical manifestations, conventional inflammatory markers such as C-reactive protein (CRP) and procalcitonin, and pathogen detection. However, these methods often have limitations in sensitivity, specificity, and early warning capability, underscoring the need for novel, high-value biomarkers to enhance diagnostic and therapeutic precision. Long pentraxin 3 (PTX3), a key acute-phase reactant protein and soluble pattern recognition receptor (PRR), has recently garnered considerable attention for its role in infectious diseases. PTX3 is rapidly synthesized by innate immune and endothelial cells in response to stimulation by pathogens or inflammatory mediators. Functionally, PTX3 contributes to host defense through opsonophagocytosis, complement activation, and modulation of inflammatory responses. Quantification of circulating PTX3 levels demonstrates potential as an adjunctive biomarker for the diagnosis of infectious diseases and holds considerable value in early risk stratification, precise disease assessment, and personalized therapeutic strategies. Nevertheless, the clinical application of PTX3 faces two major challenges in immune regulation. Its broad-spectrum responsiveness to inflammation limits its specificity in pathogen differentiation, and the mechanisms underlying its immunomodulatory activity remain complex. This review systematically summarizes recent advances in the diagnostic significance and immunoregulatory mechanisms of PTX3, providing new insights into overcoming current challenges in the precision diagnosis and treatment of infectious diseases.

## Introduction

Infectious diseases pose a substantial threat to human life and health, constituting a major global public health challenge with extensive and long-term consequences. The pathogenicity and risks of mortality and disability associated with infectious diseases have persisted throughout human history. Currently, the continuous emergence and re-emergence of novel infectious diseases worldwide continue to impose significant burdens on global public health systems and socioeconomic stability. Under these circumstances, achieving early and accurate identification, diagnosis, and effective intervention for infectious diseases has become an urgent priority requiring innovative breakthroughs.

Pentraxin 3 (PTX3), a soluble PRR, can be rapidly induced in various cell types upon stimulation by pro-inflammatory cytokines or recognition of pathogen-associated molecular patterns (PAMPs) (Massimino et al., 2023). PTX3 functions as a crucial mediator in innate immune responses against diverse pathogens including fungi, bacteria, and viruses, and contributes to the fine-tuning of inflammatory processes. Consequently, PTX3 holds substantial significance for elucidating its immunological properties, mechanisms of action, and potential clinical applications in infectious diseases.

This review aims to systematically summarize recent advances in PTX3-related research across various infectious diseases and to explore its potential future applications and research directions.

## Characteristics of PTX3

During the evolutionary development of the immune system, PRRs and immunoglobulins have demonstrated homologous evolutionary trajectories. Their soluble isoforms may represent ancestral forms of key components of adaptive immunity, providing immune protection and regulation through mechanisms such as neutralization, enhanced phagocytosis, and complement activation (Massimino et al., 2023).

PTX proteins, belonging to one of the conserved PRR families, are characterized by strong genetic conservation. PTX proteins contain a C-terminal “pentraxin domain” comprising approximately 200 amino acids and a conserved eight-amino acid sequence (His-X-Cys-X-Ser/Thr-Trp-X-Ser). Based on protein length, PTXs are classified into short-chain types such as CRP and serum amyloid P component which function as classical acute-phase proteins, and long-chain types such as PTX3 and PTX4 which serve as novel regulatory proteins (Li et al., 2023).

**Figure 1:**
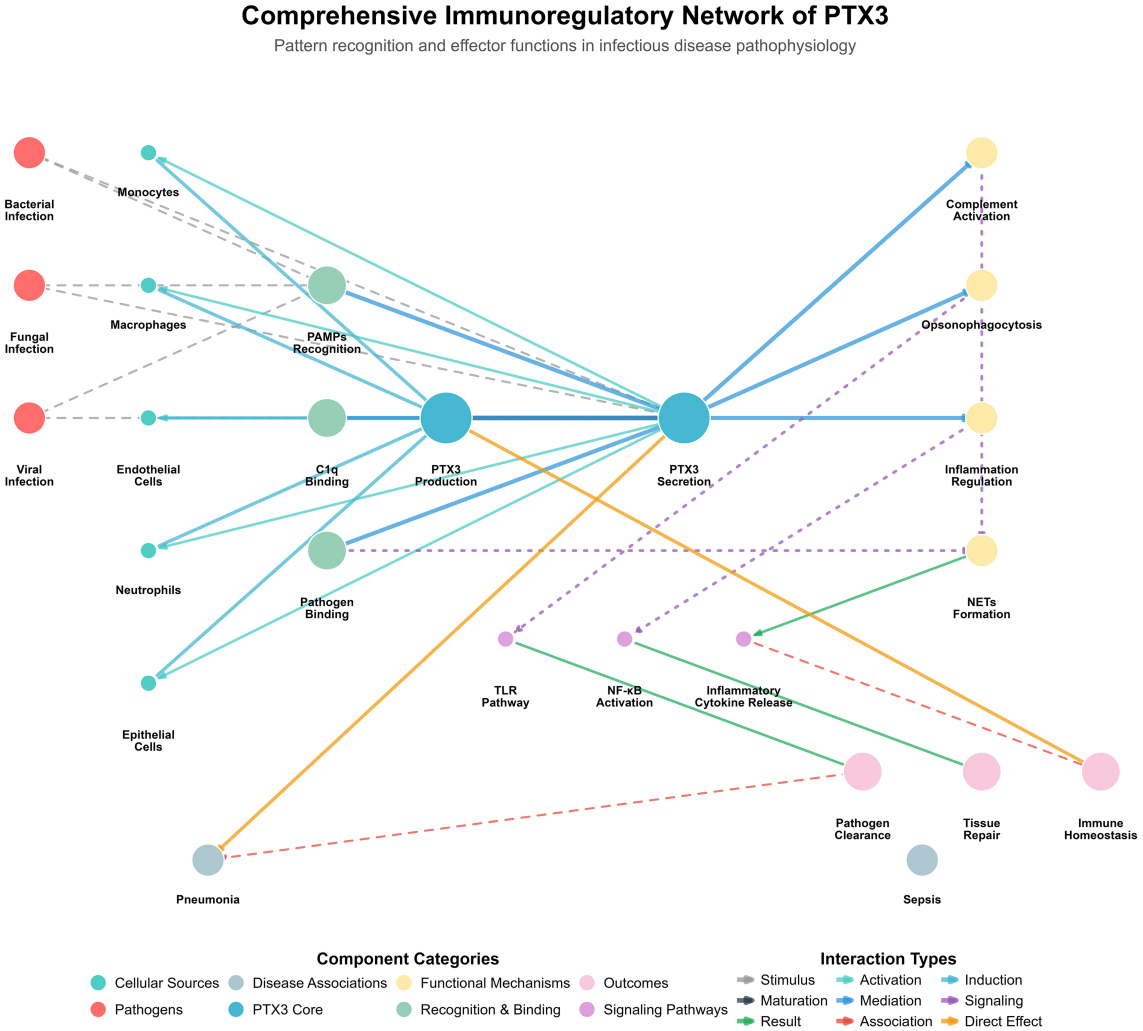
Comprehensive immunoregulatory network of PTX3 in infectious diseases. This figure illustrates the pattern recognition and effector function network of long PTX3 in the pathophysiology of infectious diseases. Various cell types (including monocytes, macrophages, endothelial cells, epithelial cells, and neutrophils) are activated upon stimulation by PAMPs via the Toll-like receptor (TLR)/nuclear factor kappa B (NF-κB) signaling pathway, leading to the production and secretion of PTX3. The secreted PTX3 acts as a central mediator that directly binds to pathogens such as bacteria, fungi, and viruses, facilitating opsonophagocytosis and pathogen clearance. Concurrently, PTX3 regulates complement activation (e.g., via binding to C1q) and fine-tunes inflammatory responses (e.g., by influencing neutrophil extracellular trap formation and inflammatory cytokine release). These intricate interactions collectively modulate immune homeostasis, impact tissue repair processes, and are associated with clinical outcomes of specific infectious diseases, such as pneumonia and sepsis. In this figure, solid lines indicate direct interactions, dashed lines indicate indirect actions or regulatory relationships, and dotted lines denote signaling events.

**Figure 2:**
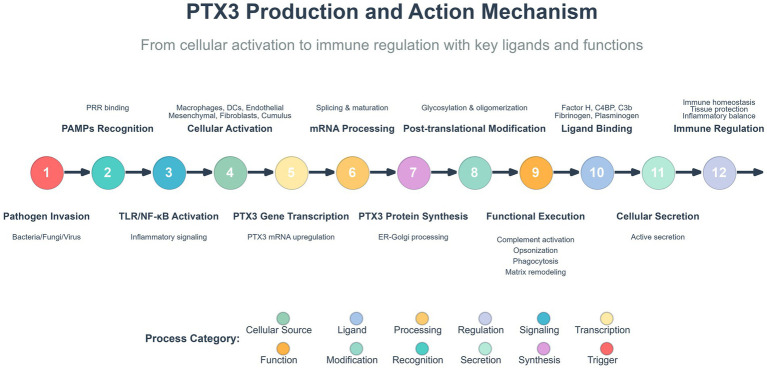
PTX3 biosynthesis, ligand interaction, and core functional network in infectious diseases. Biosynthesis and secretion: PAMPs activate innate immune cells and tissue-resident cells, upregulating PTX3 gene expression primarily via signaling pathways such as TLR/NF-κB. The synthesized PTX3 precursor undergoes post-translational modifications including glycosylation and oligomerization to form the mature octamer, which is subsequently secreted into the extracellular milieu. Molecular interactions: Secreted PTX3 functions as a multifunctional molecular hub, engaging a diverse array of ligands. These include complement system components (e.g., C1q, C4b, Factor H), various pathogens (bacteria, fungi, viruses), and extracellular matrix proteins (e.g., fibrinogen, plasminogen). Core biological functions: Through these interactions, PTX3 primarily executes three key functions: (1) Opsonization and pathogen clearance; (2) Bidirectional regulation of complement activation and inflammatory response, thereby preventing immunopathological damage; and (3) Participation in tissue protection and homeostasis maintenance. Collectively, these functions delineate the complex immunomodulatory role of PTX3 in infection.

PTX3 exhibits a highly ordered D4-symmetric octameric architecture, which serves as the molecular basis for its diverse immune functions. Its structural core is formed by eight C-terminal pentraxin domains assembled into a cube-like module responsible for ligand recognition. This core is flanked by tetrameric N-terminal coiled-coil extensions, which are connected via flexible hinge regions, conferring structural plasticity (Noone et al., 2022). The stability of this architecture relies on a dual mechanism involving salt bridges and disulfide bonds. A critical internal salt bridge network (e.g., E180-K214) guides the correct subunit assembly, while an intermolecular disulfide bond between C317 and C318 is the decisive covalent linkage joining two tetramers to form the complete octamer. Furthermore, glycosylation at the N220 site, particularly the terminal sialic acid residues, dynamically modulates its interactions with ligands such as C1q, enabling fine-tuning of its immune functions (Shah et al., 2025). In summary, the unique octameric scaffold, flexible extensions, dual stabilization system, and dynamic modifications of PTX3 collectively constitute a multifunctional platform. This sophisticated modular organization allows PTX3 to simultaneously coordinate critical biological processes, including pathogen recognition, complement activation, and inflammatory regulation. The pleiotropic immunomodulatory functions of PTX3 form a complex and integrated regulatory network in infectious diseases, which is schematically depicted in Figures 1, 2.

## PTX3 and infection-related diseases

The diagnostic value, immunological significance and clinical performance of PTX3 across different categories of infectious diseases have been extensively validated in clinical and preclinical studies, with the core findings and evidence-based data systematically summarized in Table 1.

**Table 1:** PTX3 in infectious diseases: diagnostic value and immunological significance.

Category	Disease	PTX3_levels	Diagnostic_performance	Key_findings	References
Respiratory	Community-acquired pneumonia	↑Early increase; correlates with severity	Early warning (AUC 0.796); prognosis	5.8-fold median increase (*p* < 0.0001)	Ye et al. (2020), Luo et al. (2019)
Respiratory	Invasive pulmonary aspergillosis	↑↑Highly elevated; AUC 0.999 for diagnosis	Specific diagnosis; Aspergillus vs. mucor	CC genotype OR 7.37; combined AUC 0.999	Dobiáš et al. (2022), Tang et al. (2020), Cunha et al. (2014)
Respiratory	Chronic obstructive pulmonary disease	↑Correlates with pathogen burden	Inflammation assessment	Airway microenvironment levels track pathogens	Thulborn et al. (2017)
Cardiovascular	Viral myocarditis	↑Expressed in cardiomyocytes; ∝ viral load	Viral vs. bacterial discrimination	Cardiomyocyte expression intensity ∝ viral load	Villatore et al. (2024)
Cardiovascular	Bacterial myocarditis	↑↑Strong interstitial expression	Myocardial injury marker	Strong interstitial positivity; negative controls	Sun et al. (2025), Nebuloni et al. (2011)
Digestive	Acute appendicitis	↑↑Serum levels vs. controls (*p* < 0.001)	Auxiliary diagnosis (high accuracy)	High diagnostic accuracy (non-exclusion)	Anand et al. (2022), Octavius et al. (2023)
Digestive	Severe acute pancreatitis	↑↑↑Severity and mortality correlate	Severity stratification; mortality prediction	Higher in fatal cases vs. survivors	Bao and Ge (2022), Wang et al. (2025)
Digestive	Gallbladder perforation	↑↑Perforation: 5.58 vs. 3.4 ng/mL (*p* < 0.0001)	Perforation identification	5.58 ng/mL (perforation) vs. 3.4 ng/mL (non)	Algin et al. (2019)
Urinary	Urinary tract infection	↑Secreted locally; recurrence risk marker	Recurrence risk prediction	Correlates with recurrent UTI severity	Li et al. (2025), Qiu et al. (2021)
Urinary	Hemorrhagic fever with renal syndrome	↑↑Severity correlates	Severity assessment	Peaks in early inflammatory phase	Jaillon et al. (2014)
Neurological	Bacterial meningitis	↑↑↑CSF elevation vs. viral meningitis	Bacterial vs. viral differentiation	CSF elevation superior to conventional markers	Thomsen et al. (2023), El-Kady et al. (2025)
Neurological	HTLV-1 associated myelopathy	↑Serum elevation	Potential diagnostic marker	Elevated in HTLV-1 myelopathy	Manzarinejad et al. (2023)
Other systems	Periprosthetic joint infection	↑↑↑Synovial fluid elevation	Infection diagnosis	Synovial fluid: infected vs. non-infected	Logroscino et al. (2023), Fidanza et al. (2025)
Other systems	Cutaneous leishmaniasis	↑Local lesion elevation	Immune response assessment	PTX3-deficient mice: reduced lesions/parasites	Gupta et al. (2020)

Arrows indicate relative PTX3 levels: ↑, mild increase; ↑↑, moderate increase; ↑↑↑, strong increase. AUC, area under the curve; CSF, cerebrospinal fluid; HTLV-1, human T-lymphotropic virus type 1; OR, odds ratio; UTI, urinary tract infection.

## Role in immune regulation and clinical significance of PTX3 in pulmonary infections

PTX3 can rapidly form complexes within pulmonary infection foci as early as 4–6 h after infection, with its biosynthesis synergistically driven by pathogen-stimulated vascular endothelial cells, alveolar epithelial cells, and immune effector cells (Madkour et al., 2024). This process demonstrates particular clinical significance in coronavirus disease 2019 (Barbosa et al., 2024; Assandri et al., 2022). PTX3 not only serves as a specific biomarker for disease staging but also enables effective prediction of secondary infection risk and clinical outcomes. Compared with conventional inflammatory markers, the unique temporal responsiveness of PTX3 provides novel insights into optimizing intensive care strategies and guiding the precise application of antimicrobial agents (Scavello et al., 2024; Lapadula et al., 2022; Feitosa et al., 2023).

Proteomic studies have revealed that PTX3 recognizes influenza viruses by binding to viral surface glycoproteins such as hemagglutinin (HA) and neuraminidase (NA), with notable subtype-specific variations in its binding affinity. For instance, PTX3 exhibits stronger binding activity toward the H3N2 subtype, while its interaction with H1N1 strains is comparatively weaker (Garlanda et al., 2002).

In bacterial pneumonia, PTX3 exhibits pathogen-specific characteristics. Genetic knockout of PTX3 attenuates inflammatory responses and improves survival rates in *Streptococcus pneumoniae* infection models compared with control groups (Koh et al., 2017). Conversely, in *Pseudomonas aeruginosa* infection models, the loss of PTX3 produces opposite outcomes (He et al., 2007). This paradox suggests that the functional role of PTX3 may be dependent on the infecting pathogen. Recent studies have further revealed that PTX3 mitigates inflammatory damage and bacterial dissemination during invasive *S. pneumoniae* infection by inhibiting P-selectin-dependent neutrophil recruitment. PTX3-deficient mice exhibit higher bacterial loads, enhanced neutrophil infiltration, and increased mortality. Moreover, human PTX3 gene polymorphisms (e.g., rs3816527) are also associated with the risk of invasive pneumococcal infection (Porte et al., 2023).

In patients with chronic obstructive pulmonary disease (COPD), dynamic correlations have been observed between PTX3 concentrations in the airway microenvironment and pathogen burden (Thulborn et al., 2017). Analyses indicate that peripheral blood PTX3 levels are significantly and positively correlated with bacterial load, regardless of whether patients with COPD are in a stable phase or experiencing an acute exacerbation. However, it must be emphasized that for patients with COPD, lower respiratory tract specimens (e.g., sputum or bronchoalveolar lavage fluid) provide a more accurate reflection of localized bacterial infection. Blood samples may fail to detect local colonization or early invasive infection.

Beyond acute bacterial infections, genetic polymorphisms of PTX3 also demonstrate significant relevance in chronic infections. Specifically, evidence indicates that the PTX3 haplotype G-A-G is associated with increased resistance to tuberculosis infection (Olesen et al., 2007). During disease progression monitoring, PTX3 levels significantly decline after 8 weeks of treatment in patients with tuberculosis (Sigal et al., 2017). This dynamic alteration highlights the critical role of PTX3 in evaluating disease severity and monitoring therapeutic response in tuberculosis. Genetic testing of blood samples can be utilized to identify susceptible individuals and guide prophylactic monitoring.

In fungal pneumonia, bronchoalveolar lavage remains the gold-standard method for early diagnosis in patients with suspected invasive fungal disease, particularly among immunocompromised patients (Dobiáš et al., 2022). A clinical study demonstrated that measuring PTX3 levels in bronchoalveolar lavage fluid can effectively distinguish invasive aspergillosis (IA) from chronic pulmonary aspergillosis, with an area under the curve (AUC) of 0.999 (Dobiáš et al., 2022). Furthermore, when combined with the detection of fungal siderophores, this measurement enables rapid differentiation between *Aspergillus* and *Mucor* species infections. Polymorphism at the rs3816527 locus further confirms the association between PTX3 and susceptibility to fungal infection, with the CC genotype significantly increasing the risk of IA (odds ratio = 7.37) (Tang et al., 2020). Furthermore, genetic deficiency in PTX3 directly impairs the host’s antifungal defense. A prospective study in patients undergoing hematopoietic stem cell transplantation (HSCT) identified that donor carriage of the homozygous h2/h2 haplotype of the PTX3 gene was an independent risk factor for invasive aspergillosis in recipients (discovery cohort: adjusted hazard ratio HR = 3.08, *p* = 0.003; validation cohort: adjusted odds ratio OR = 2.78, *p* = 0.03). Mechanistic studies revealed that the h2/h2 haplotype leads to decreased stability of PTX3 mRNA, resulting in deficient PTX3 expression in neutrophils and consequently impairing their ability to phagocytose and clear *Aspergillus fumigatus* conidia. This study not only genetically confirms the critical role of PTX3 in anti-Aspergillus immunity but also provides a potential genetic marker for risk stratification in high-risk patients (Cunha et al., 2014).

Regarding inflammation assessment in community-acquired pneumonia (CAP), PTX3 demonstrates superior temporal sensitivity compared with conventional inflammatory markers such as CRP, particularly during early-stage disease monitoring (Ye et al., 2020).

Notably, PTX3 also exhibits strong prognostic value for severe disease. The median concentration of PTX3 increases by 5.8-fold in severe cases compared with mild cases (*p* < 0.0001) (Luo et al., 2019), effectively predicting 30-day survival outcomes in patients with severe CAP (AUC = 0.796). In patients with CAP, blood testing allows for rapid assessment of systemic inflammatory response and prognosis, obviating the need for routine bronchoalveolar lavage (Luo et al., 2019).

In summary, measurement of PTX3 concentration in bronchoalveolar lavage fluid remains indispensable for achieving early and precise diagnosis in cases of invasive fungal disease, infections in immunocompromised patients, and situations with an unidentified pathogen. In contrast, detection of PTX3 levels in blood samples is suitable for the assessment of systemic infection, severity stratification in CAP, and screening of high-risk populations, offering operational simplicity and utility for early risk evaluation. Therefore, in severe or complex infections, combined testing of bronchoalveolar lavage fluid and blood samples can enhance both the comprehensiveness and timeliness of diagnosis. Due to its early responsiveness, pathogen-specific interactions, and dynamic serum changes, PTX3 represents a promising biomarker for the identification and prognostic assessment of pulmonary infections.

## Research progress of PTX3 in infectious myocarditis

There is an urgent need for non-invasive, early-warning biomarkers capable of identifying the risk of myocarditis progression to dilated cardiomyopathy. As a multifunctional regulatory protein involved in inflammation, PTX3 demonstrates potential value in infection-related cardiovascular injury through three major mechanisms: pathogen recognition, regulation of inflammatory balance, and promotion of tissue repair. Notably, in murine models of sepsis-induced cardiomyopathy, PTX3 has been identified as a critical component of the NLRP3 inflammasome. Its pathological upregulation directly triggers cardiomyocyte pyroptosis, leading to cardiac dysfunction. This finding underscores the potent pro-inflammatory and injury-inducing role of PTX3 within specific infectious contexts (Sun et al., 2025).

Research has shown that in pathological cardiac tissues from patients with infectious myocarditis caused by diverse pathogens, including fungi, bacteria, protozoa, and *Mycobacterium tuberculosis*, PTX3 exhibits significant expression specificity.

Strong positivity is observed in the interstitium, whereas no expression is detected in normal cardiac tissues (Nebuloni et al., 2011). Notably, studies have revealed that in viral myocarditis, PTX3 is localized within cardiomyocytes, and its expression intensity directly correlates with viral load (Villatore et al., 2024). These differential findings regarding expression localization (intracardiomyocytic vs. interstitial) and primary cellular sources (cardiomyocytes vs. non-cardiomyocytes) may provide valuable reference criteria for distinguishing between pathogen types. PTX3 exhibits pathogen-specific expression patterns and demonstrates clear cardioprotective effects in infectious myocarditis, indicating its potential as a biomarker for risk stratification and a therapeutic target. Nevertheless, further research is required to elucidate the underlying regulatory mechanisms across different infection types and to validate its diagnostic specificity.

## Expression profile of PTX3 in digestive system infections and non-infectious inflammation

Although some digestive system disorders are not pathognomonic infectious diseases, PTX3, as an acute-phase protein, can nevertheless provide valuable reference information for differentiating inflammatory states and assessing their severity.

PTX3 has garnered attention due to its characteristic prompt elevation following the onset of inflammation. In acute digestive emergencies where infection may be involved, PTX3 demonstrates diagnostic value. Studies have demonstrated that serum PTX3 levels in patients with acute appendicitis (AA) are significantly higher than those in both healthy controls and individuals with non-specific abdominal pain (Anand et al., 2022). Furthermore, Octavius et al. confirmed that PTX3 demonstrates high diagnostic accuracy for AA, with superior sensitivity and specificity (Octavius et al., 2023). However, subsequent research indicates that, as a non-specific acute-phase reactant that can increase under various inflammatory conditions, PTX3 alone cannot effectively exclude other causes of acute abdominal pain. Therefore, it may not serve as a reliable biomarker for the differential diagnosis of AA. PTX3 also demonstrates diagnostic value in differentiating gallbladder perforation. One study reported that the median PTX3 concentration was 5.58 ng/mL in patients with gallbladder perforation compared to 3.4 ng/mL in those without, representing a statistically significant difference (*p* < 0.0001) (Algin et al., 2019).

PTX3 also plays a significant role in typical sterile inflammatory conditions of the digestive system. In pancreatitis, PTX3 has also shown potential clinical relevance. Serum PTX3 levels were found to be higher in patients with severe acute pancreatitis (SAP) than in those with non-severe forms. Moreover, PTX3 concentrations were significantly elevated in deceased patients with SAP compared to survivors, suggesting a correlation between serum PTX3 levels and disease severity (Bao and Ge, 2022). Wang et al. (2025) further revealed that PTX3 disrupts oxidative phosphorylation by inhibiting the electron transport chain, ultimately leading to mitochondrial dysfunction and pancreatic acinar cell injury, thereby providing a theoretical foundation for targeting the PTX3 signaling pathway. In specific liver diseases such as alcoholic hepatitis, PTX3 demonstrates significant immunomodulatory and prognostic evaluation value. Patients with alcoholic hepatitis exhibit markedly elevated PTX3 expression, which correlates with disease severity, endotoxemia, and short-term mortality. Experimental studies indicate that PTX3 is primarily produced by activated hepatic stellate cells and attenuates LPS-induced liver injury by modulating TLR4 signaling and macrophage polarization, suggesting its dual role as both a biomarker and an endogenous protective factor (Perea et al., 2017).

Additionally, PTX3 plays a role in digestive system complications arising from systemic infections. A study by Wang et al. (2024) found that PTX3 deficiency attenuated LPS-induced liver injury and improved survival in mice by promoting hepatocyte ferroptosis through upregulation of TFRC expression and recruiting M1-type macrophages via the CCL20/CCR6 axis. This finding suggests an organ-specific immunomodulatory function for PTX3 during systemic infection, presenting a novel therapeutic target for sepsis-associated liver injury.

In summary, PTX3 possesses diagnostic potential within the spectrum of digestive system inflammatory diseases. Nevertheless, owing to its broad-spectrum inflammatory responsiveness and limited specificity in distinguishing different subtypes of appendicitis, further research is warranted to elucidate the distinct regulatory mechanisms of PTX3 across various digestive infections. Integrating PTX3 assessment with other complementary biomarkers may enhance diagnostic precision and clinical applicability.

## PTX3 and urinary infections

Urinary tract infection (UTI) is a common and recurrent clinical condition, primarily caused by pathogens such as *Escherichia coli*, *Klebsiella* spp., *Enterococcus* spp., and *Pseudomonas* spp. that migrate from the rectal and perineal regions to colonize the urinary tract (Sabih and Leslie, 2024). During the pathological progression of UTI, various stimulatory factors can induce cells within the urinary tract such as urothelial cells and proximal tubular epithelial cells to secrete PTX3 (Li et al., 2025). PTX3 plays multiple essential roles in host defense (Qiu et al., 2021). On one hand, a portion of PTX3 participates in the formation of extracellular DNA networks, such as neutrophil extracellular traps, which may represent one mechanism by which PTX3 contributes to innate immunity. On the other hand, another portion of PTX3 inhibits the interaction between neutrophils and P-selectin. More importantly, PTX3 can directly bind to invading pathogens such as *E. coli*. These interactions collectively facilitate microbial recognition, subsequent phagocytosis, phagosome maturation, and activation of the complement cascade. Furthermore, PTX3 exerts negative feedback regulation by modulating the extent of complement activation and immune cell recruitment, thereby preventing tissue injury resulting from excessive immune responses. PTX3 has also been investigated as a potential biomarker for UTI.

Studies have demonstrated that PTX3 levels correlate with both the severity and recurrence risk of recurrent UTI (RUTI) (Qiu et al., 2021), suggesting that PTX3 may serve as a predictive marker for RUTI progression. In patients with hemorrhagic fever with renal syndrome, Du et al. (2021) observed that serum PTX3 concentrations increase in parallel with disease severity. In patients with acute pyelonephritis, significantly elevated levels of PTX3 have been detected in both urine and serum. Its concentration correlates with the severity of infection and decreases following effective antibiotic therapy (Jaillon et al., 2014). This finding highlights the diagnostic and monitoring potential of PTX3 in UTIs.

Furthermore, PTX3 levels exhibit significant correlations with hospitalization duration and conventional inflammatory markers; however, no direct correlation has been observed with renal function indicators such as blood urea nitrogen or creatinine. Notably, the dynamic peak of PTX3 concentration predominantly occurs during the early phase of inflammation (Du et al., 2021).

Thus, PTX3 plays a direct role in host defense and tissue protection by facilitating pathogen recognition and clearance while finely regulating immune responses. The expression level of PTX3 can provide valuable insights into infection severity. However, discrepancies between PTX3 levels and renal dysfunction, along with its stage-specific expression dynamics, underscore the need for cautious clinical interpretation aligned with the stage of disease progression. Therefore, a comprehensive clinical assessment should incorporate PTX3 evaluation along with conventional renal function markers to enhance diagnostic accuracy and reliability.

## PTX3 and neurological infections

In central nervous system (CNS) infections, PTX3 can be induced in neurons, microglial cells, and astrocytes (Manzarinejad et al., 2023). Studies have demonstrated that PTX3 levels in cerebrospinal fluid (CSF) are significantly higher in patients with bacterial meningitis than in those with viral meningitis, viral encephalitis, or Lyme neuroborreliosis (Thomsen et al., 2023). This finding suggests that PTX3 can effectively differentiate bacterial meningitis from other CNS infections. Moreover, PTX3 exhibits superior diagnostic performance for bacterial meningitis compared with conventional biochemical markers such as CRP (El-Kady et al., 2025). Thomsen et al. (2023) further observed that PTX3 is typically undetectable or present at very low levels in the CSF of patients without meningitis. This observation implies that in the absence of overt CNS inflammatory responses or disruption of the blood–brain barrier (BBB), PTX3 does not readily cross from the bloodstream into the CSF. Therefore, the diagnostic utility of PTX3 for neurological diseases may be limited. Additionally, PTX3 shows correlations with certain viral CNS infections. Manzarinejad et al. (2023) reported that serum PTX3 levels are elevated in patients with human T-cell leukemia virus type 1 (HTLV-1)-associated myelopathy, suggesting its potential as a diagnostic marker for this condition.

Hence, the diagnostic application of PTX3 in CNS infections requires both BBB disruption and activation of local CNS inflammation. Elevated serum PTX3 levels should therefore be interpreted with caution and always in conjunction with clinical findings. Although PTX3 demonstrates considerable value in differentiating bacterial meningitis from other CNS infections, limitations persist due to uncertainty regarding its cellular source and the potential risk of misdiagnosis.

## PTX3 in orthopedic infections

PTX3 is a significant component of the bone microenvironment, expressed by bone progenitor cells and osteoblasts, and plays a role in maintaining bone homeostasis and facilitating fracture healing (Parente et al., 2019). It demonstrates clear diagnostic value in orthopedic infections. Studies have confirmed that the concentration of PTX3 in the synovial fluid of patients with periprosthetic joint infection (PJI) of the hip or knee is significantly higher than in patients undergoing aseptic revision. This marker shows a diagnostic specificity of up to 97% and an AUC of 0.96 (Logroscino et al., 2023). Furthermore, serum PTX3 levels rise rapidly in the early postoperative period (4–6 h) and normalize swiftly with the resolution of inflammation, demonstrating a more sensitive dynamic trend compared to markers like the erythrocyte sedimentation rate (ESR) (Fidanza et al., 2025). Genetic analysis further reveals a strong positive correlation between synovial fluid PTX3 levels and IL-1β concentration (Spearman *r* = 0.67, *p* = 0.004). Moreover, PTX3 levels are significantly associated with IL-1β gene polymorphisms (e.g., rs2853550), uncovering its regulatory mechanism within the local infectious response (Granata et al., 2024). In summary, PTX3 is not only a key regulatory protein in bone metabolism but also a highly specific diagnostic biomarker for PJI.

## PTX3 and skin infections

PTX3 play an essential regulatory role in immune responses during skin infections. However, its immunomodulatory effects appear paradoxical across different disease contexts. In a cutaneous leishmaniasis model, local PTX3 expression in lesions was markedly upregulated, yet PTX3-deficient mice exhibited smaller lesions, reduced parasite loads, and abnormally elevated interleukin-17A levels (Gupta et al., 2020). Similarly, in leprosy, patients presented increased serum PTX3 concentrations that declined following the initiation of multidrug therapy (Moraes et al., 2023). Notably, PTX3 levels differed between patients with type I (reversal reaction) and type II (erythema nodosum leprosum) reactions, with higher levels observed in type II cases. These findings indicate that, beyond its diagnostic potential in leprosy, PTX3 may also contribute to disease subtype differentiation.

## PTX3 in sepsis and septic shock

Sepsis is a life-threatening condition defined as organ dysfunction caused by a dysregulated host response to infection. The early identification of sepsis and accurate assessment of its severity and prognosis are paramount for clinical management. PTX3 has emerged as a biomarker of significant value in the stratification of sepsis and septic shock.

A systematic review and meta-analysis by Lee et al., encompassing 16 studies and 3,001 patients, demonstrated that PTX3 levels were significantly elevated in patients with more severe sepsis, particularly in septic shock (standardized mean difference 18.5 ng/mL). Furthermore, non-survivors had significantly higher PTX3 levels compared to survivors. Pooled analysis indicated that elevated PTX3 was associated with an approximately two-fold increased risk of all-cause mortality (HR 1.91, 95% CI 1.53–2.46), underscoring its potential as a robust biomarker for assessing disease severity and predicting mortality risk in sepsis (Lee et al., 2018).

The ALBIOS trial represents one of the largest studies investigating the clinical utility of PTX3 in severe sepsis and septic shock. By serially measuring plasma PTX3 levels at days 1, 2, and 7 in 958 patients, the study provided comprehensive insights. Key findings included: (1) Markedly elevated baseline PTX3 levels (median 72 ng/mL), which correlated positively with the number and severity of organ dysfunctions; (2) High early PTX3 levels predicted the subsequent development of new organ failures; and (3) Most importantly, the dynamic change of PTX3 carried critical prognostic information—a smaller decrease in PTX3 levels over time was associated with a higher 90-day mortality risk. Notably, within the septic shock subgroup, patients receiving albumin supplementation exhibited significantly lower PTX3 levels compared to those receiving crystalloids alone, suggesting that albumin may modulate PTX3 through anti-inflammatory effects or direct binding. This observation offers a novel perspective for immunomodulatory therapy in sepsis (Caironi et al., 2017).

In summary, PTX3 serves not only as a marker reflecting the initial severity of the septic insult but also, through its kinetic profile, as a powerful tool for prognostic evaluation and potentially for monitoring therapeutic response.

## Laboratory detection methods

The quantitative measurement of PTX3 in clinical and research settings primarily relies on immunoassays based on antigen–antibody reactions, such as Enzyme-Linked Immunosorbent Assay (ELISA) or Chemiluminescence Immunoassay (CLIA), performed on serum or plasma samples. These highly sensitive methods provide the essential technical foundation for investigating the role of PTX3 in sepsis severity assessment and outcome prediction (Riedel and Carroll, 2013). However, it is important to note that despite its promising research value, the measurement of PTX3 remains predominantly within the research domain and has not yet been adopted as a routine diagnostic test in clinical practice.

## Conclusion

PTX3, as a PRR, modulates host immunity through dual mechanisms. It mediates pathogen recognition across bacteria, fungi, and viruses, while simultaneously maintaining immune homeostasis by regulating complement activation and inflammatory responses. Its multifaceted roles in innate immunity, tissue repair, and inflammation regulation underscore its potential as both a biomarker and a therapeutic target (Garlanda et al., 2018). Serum PTX3 levels demonstrate strong correlations with disease severity in conditions such as pneumonia, UTI, and infectious myocarditis, offering valuable insights for precise diagnosis, risk stratification, and prognostic evaluation in specific infectious diseases. Nevertheless, further research is necessary to establish standardized diagnostic criteria for PTX3 and to explore its potential as a therapeutic target. Future breakthroughs are expected through multicenter cohort validation and mechanism-based interventional studies.

## Glossary

PTX3: pentraxin 3
PRR: pattern recognition receptor
PAMPs: pathogen-associated molecular patterns
CRP: C-reactive protein
COPD: chronic obstructive pulmonary disease
IA: invasive aspergillosis
AUC: area under the curve
CAP: community-acquired pneumonia
AA: acute appendicitis
SAP: severe acute pancreatitis
UTI: urinary tract infection
CNS: central nervous system
CSF: cerebrospinal fluid
BBB: blood-brain barrier
HTLV-1: human T-cell leukemia virus type 1
PJI: periprosthetic joint infection
ESR: erythrocyte sedimentation rate
RUTI: recurrent urinary tract infection
HSCT: hematopoietic stem cell transplantation
HA: hemagglutinin
NA: neuraminidase
ELISA: enzyme-linked immunosorbent assay
CLIA: chemiluminescence immunoassay
NLRP3: NOD-, LRR- and pyrin domain-containing protein 3
LPS: lipopolysaccharide
TLR4: toll-like receptor 4
TFRC, CD71: transferrin receptor
CCL20: C-C motif chemokine ligand 20
CCR6: C-C motif chemokine receptor 6
IL-1β: interleukin-1 beta
HR: hazard ratio
OR: odds ratio
CI: confidence interval
