# Correction: Diagnostic value and challenges in the immune regulation of pentraxin 3 in infectious diseases

**DOI:** 10.3389/fmicb.2026.1819193

**Published:** 2026-03-17

**Authors:** Frontiers Production Office

**Affiliations:** Frontiers Media SA, Lausanne, Switzerland

**Keywords:** biomarker, immune regulation, infectious diseases, inflammatory response, PTX3

Affiliation [Department of Intensive Care Unit, Affiliated Hospital of Hebei University, Baoding, China] was omitted from the paper. This affiliation has now been added, and the assigned affiliations for all authors have been corrected. The corrected author list and affiliation list appears below.

Teng-Hao Shao^1†^, Tie-Min Li^2†^, Jin-Wen Zhang^3^, Xiao-Wei Lv^1^, Xin-Tong Li^3^, Na Cui^1*^ and Ping Sheng^1*^

^1^Department of Intensive Care Unit, Affiliated Hospital of Hebei University, Baoding, China

^2^Department of Laboratory Medicine, Affiliated Hospital of Hebei University, Baoding, China

^3^Health Science Center, Hebei University, Baoding, Hebei, China

The original version of this article has been updated.

